# Limited penetrance of dominantly inherited *AIRE* variants in a population-based cohort

**DOI:** 10.1093/hmg/ddag047

**Published:** 2026-06-09

**Authors:** Suraj N Ramchand, Jacques Murray Leech, Luke N Sharp, Georgia Bonfield, Amber M Luckett, Michael N Weedon, Kashyap A Patel, Gareth Hawkes, Matthew B Johnson

**Affiliations:** Department of Clinical and Biomedical Sciences, University of Exeter, Barrack Road, Exeter, Devon, EX2 5NA, United Kingdom; Department of Clinical and Biomedical Sciences, University of Exeter, Barrack Road, Exeter, Devon, EX2 5NA, United Kingdom; Department of Clinical and Biomedical Sciences, University of Exeter, Barrack Road, Exeter, Devon, EX2 5NA, United Kingdom; Department of Clinical and Biomedical Sciences, University of Exeter, Barrack Road, Exeter, Devon, EX2 5NA, United Kingdom; Department of Clinical and Biomedical Sciences, University of Exeter, Barrack Road, Exeter, Devon, EX2 5NA, United Kingdom; Department of Clinical and Biomedical Sciences, University of Exeter, Barrack Road, Exeter, Devon, EX2 5NA, United Kingdom; Department of Clinical and Biomedical Sciences, University of Exeter, Barrack Road, Exeter, Devon, EX2 5NA, United Kingdom; Department of Clinical and Biomedical Sciences, University of Exeter, Barrack Road, Exeter, Devon, EX2 5NA, United Kingdom; Department of Clinical and Biomedical Sciences, University of Exeter, Barrack Road, Exeter, Devon, EX2 5NA, United Kingdom

**Keywords:** *AIRE*, dominant-negative, autoimmunity, rare variants, UK Biobank

## Abstract

Pathogenic variants in the autoimmune regulator gene (*AIRE*) cause Autoimmune polyendocrine syndrome type 1 (APS-1). The majority of disease-causing variants are inherited recessively, while several heterozygous *AIRE* variants have been reported in the literature as having dominant-negative or partially inhibitory effects. Additionally, some heterozygous *AIRE* variants have been implicated in milder and more common autoimmune phenotypes in disease cohort studies. The contribution of these variants to disease risk in unselected populations has not been assessed at scale. We aimed to investigate the prevalence and phenotypic impact of rare coding variants in *AIRE* in 449 075 participants from the UK Biobank (UKB). A literature review identified 25 rare heterozygous *AIRE* variants previously reported in association with autoimmunity, including variants described as dominant-negative or partially inhibitory, 13 of which were observed in UKB, collectively carried by 2123 participants. Filtering and annotation of rare coding *AIRE* variants identified a further 180 rare coding *AIRE* variants in 1934 UKB participants. Phenotype association analysis revealed no significant associations between rare heterozygous *AIRE* variants previously reported in association with autoimmunity and APS-1-associated traits after multiple-testing correction. Similarly, no statistically significant associations were observed for other heterozygous carriers of missense variants, frameshift/protein-truncating variants, or domain-specific groupings. These findings are consistent with a predominantly recessive model of *AIRE*-associated disease and indicate limited penetrance of rare heterozygous variants in the UKB population. Our work highlights the need for cautious interpretation of heterozygous *AIRE* variants in clinical settings.

## Introduction

Autoimmune polyendocrine syndrome type 1 (APS-1, also known as APECED, OMIM #240300) is a rare childhood-onset disorder caused by biallelic loss-of-function mutations in the autoimmune regulator (*AIRE*) gene [1–3]. It is clinically defined by the presence of two of three major disease components: primary adrenal insufficiency, hypoparathyroidism, and chronic mucocutaneous candidiasis [3, 4], although the clinical spectrum includes a myriad of other features [5–7]. *AIRE* encodes a transcriptional regulator in medullary thymic epithelial cells [8] that enforces central immune tolerance by driving expression of tissue-restricted antigens and facilitating deletion of autoreactive T cells [9, 10]. The AIRE protein comprises a caspase activation and recruitment domain (CARD) implicated in oligomerisation, a second domain (SAND) involved in DNA binding, and two plant homeodomain zinc fingers (PHD1, PHD2) that act as histone-recognition modules, features that together organise AIRE’s chromatin engagement and transcriptional activity.

While APS-1 is classically autosomal recessive, three heterozygous *AIRE* variants have been reported as having dominant-negative (DN) effects and segregating with non-classical autoimmune phenotypes in families. In particular, NM_000383.4:c.682G > T (p.Gly228Trp) and NM_000383.4:c.932G > A (p.Cys311Tyr) have relatively strong supporting evidence from family co-segregation, *in vitro* functional studies and *in vivo* models [11–16]. In addition, NM_000383.4:c.1336 T > G (p.Cys446Gly) has supporting *in vivo* evidence, although the reported phenotype is milder and has variable penetrance [12, 16]. Similarly, a broader set of rare heterozygous *AIRE* variants previously reported in association with autoimmunity have been described as showing DN-like or partial inhibitory effects in model systems and have been reported to have milder, organ-specific, or later-onset autoimmunity with incomplete penetrance and variable expressivity [11–13]. Many of these literature-reported heterozygous variants cluster within the PHD1 domain, consistent with their roles in recognising H3K4me0 and anchoring AIRE to chromatin. However, the clinical relevance of these mutations remains unclear, particularly in unselected populations, and there are limitations with *in vitro* approaches to assessing the impact of genetic variants, as they may not reflect biological significance at the organism level [16, 17]. These variants are rare in the general population and are most often observed in clinically ascertained families or isolated individuals [11–13], limiting inference about population-level risk [18, 19]. Their phenotypic effects also vary widely—even within families—and may be influenced by modifier genes or environmental exposures, making their pathogenicity difficult to assess [18, 19].

To clarify the contribution of these variants to autoimmune disease risk, we investigated the prevalence and phenotypic impact of rare heterozygous *AIRE* variants using whole-genome sequencing from 502 154 individuals, of whom 449 075 have linked healthcare records in UK Biobank (UKB) [20].

## Results

### Identification of 4057 rare *AIRE* variant carriers in UKB

**Figure 1:**
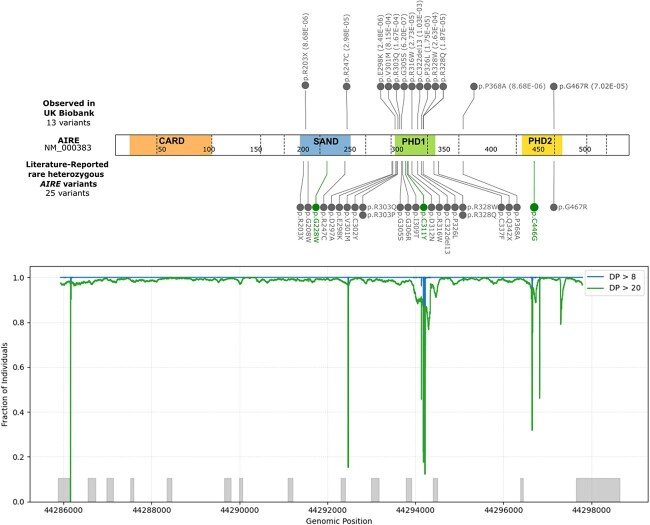
Distribution and coverage of literature-reported heterozygous *AIRE* variants across functional domains. Top panel: Schematic representation of the *AIRE* gene structure showing literature-reported heterozygous *AIRE* variants and those observed in UKB. Variants with in vivo or strong familial segregation support (highlighted in green). All remaining variants are shown in grey and represent rare heterozygous *AIRE* variants previously reported in association with mild, later-onset autoimmunity in the clinical literature. Functional domains include CARD, SAND, PHD1, and PHD2. Bottom panel: Whole-genome sequencing depth of coverage from UKB genomes across the *AIRE* gene locus. The Y-axis represents the fraction of individuals with read depth > 8 and > 20 at each genomic position (X-axis). Bars across the X-axis indicate the location of exons across the gene.

Across 449 075 UKB participants, we identified 2123 carriers of 13 of the 25 rare heterozygous *AIRE* variants. Sequencing coverage across the interrogated regions and functional domains was high (Fig. 1), and the variant and cohort breakdown is provided in Supplementary Fig. 1. Notably, the three variants with in vivo or familial co-segregation support: p.Cys311Tyr, p.Gly228Trp and p.Cys446Gly, were absent from UKB, as were several other reported heterozygous variants, including p.Cys337Phe. The partially inhibitory deletion NM_000383.4:c.967_979del13bp (p.Leu323Serfs^*^51) with 1180 carriers was the most frequent variant in this group. Amongst missense variants in this group, the most common were NM_000383.4:c.901G > A (p.Val301Met) and NM_000383.4:c.982C > T (p.Arg328Trp), with 481 and 226 individuals respectively. In total, nine literature-reported variants were identified in 2111 carriers. The remaining 12 individuals carried one of the four variants with a MAC < 10 and were therefore collapsed into the ultra-rare bin for genetic burden testing (Supplementary Table 2). Beyond this literature-reported set, we aggregated 180 additional rare coding *AIRE* variants carried by 1934 individuals and analysed them by predicted consequence and by domain (Supplementary Fig. 2).

### Rare heterozygous *AIRE* variants are not associated with autoimmune disease risk

After correction for multiple testing (FDR at 5%), we found no statistically significant associations between literature-reported heterozygous *AIRE* variants and autoimmune phenotypes; all adjusted p-values (q) were ≥ 0.99. Several nominal associations (*P* < 5 × 10^−2^) were observed, including increased odds of cholelithiasis in carriers of ultra-rare literature-reported variants (odds ratio [OR] = 4.95, 95% CI [1.34–18.28], P = 3.60 × 10^−2^), but none survived FDR correction.

**Figure 2:**
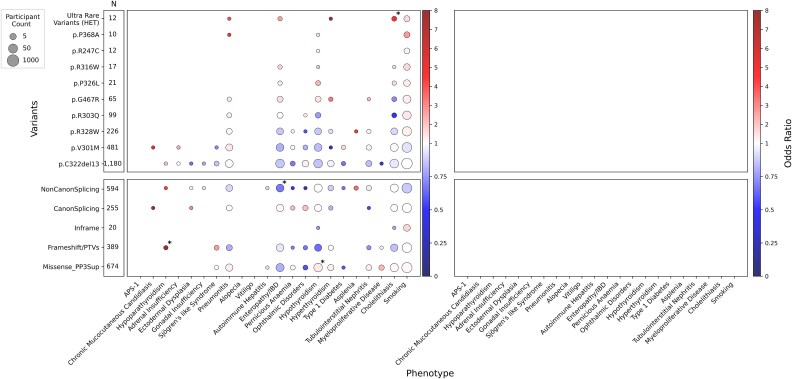
Phenotypic associations of literature-reported heterozygous *AIRE* variants and functional classes in the UKB. Left panel: Association analysis (uncorrected) between *AIRE* variants and a broad range of autoimmune and related phenotypes. Each bubble represents the OR for a given variant (rows) and phenotype (columns). Bubble size corresponds to the number of participants carrying the variant with the phenotype, and colour indicates the OR (red = OR > 1; blue = OR < 1). Asterisks (^*^) indicate nominally significant associations (*P* < 0.05). Variants are grouped into two panels: Top—Literature-reported heterozygous *AIRE* variants (e.g. p.V301M, p.R328W, p.G467R); Bottom—Aggregated rare coding variant classes based on predicted consequence and in-silico predictors (including AlphaMissense supportive pathogenic variants, frameshift/protein-truncating variants, and splice-site variants). Right panel: FDR-adjusted p-values for the same associations. No associations remained statistically significant after multiple testing correction. Phenotypes along the x-axis include both classic autoimmune conditions (e.g. vitiligo, Addison’s disease, APS-1 features) and related traits. Nominal associations should be interpreted cautiously, given the limited carrier count and the absence of significance after multiple testing correction.

Analysis of other rare coding variant classes—including missense, frameshift/protein-truncating variants (PTVs, excluding those in the terminal exon or the last 50 bp of the penultimate exon, and therefore not expected to result in nonsense-mediated decay and loss of the allele), in-frame indels, canonical and non-canonical splice-site variants did not yield statistically significant associations with autoimmune phenotypes (Fig. 2). A few nominal effects were observed, including an OR of 13.05 (95% CI [3.22–52.76], *P* = 1.09 × 10^−2^) for hypoparathyroidism among frameshift/PTV carriers, an OR of 1.43 (95% CI [1.10–1.86], *P* = 1.01 × 10^−2^) for hypothyroidism among carriers of a missense variant with supportive computational evidence and a protective OR of 0.67 (95% CI [0.46–0.98], *P* = 3.70 × 10^−2^) for enteropathies and malabsorption disorders in non-canonical splice variant carriers. These nominal associations did not survive FDR correction (q ≥ 0.99) and should be interpreted cautiously.

To validate our findings, we used existing phenome-wide association studies based on UKB data. There were no significant associations between *AIRE* missense/PTVs and immune-related traits in either the AZPheWAS [21] or GeneBass [22] databases. No significant associations were observed, although two were close to the suggestive significance threshold of *P* = 1 × 10^−6^ (*Staphylococcal arthritis and polyarthritis*, *P* = 7.44 × 10^−6^; *Iron deficiency anaemia, unspecified*, *P* = 8.45 × 10^−6^), likely reflecting false positives given the established role of *AIRE* in central tolerance [23]. Together, these findings suggest that heterozygous coding variation in *AIRE* does not act as a strong, independent risk factor for autoimmunity in the general population.

Additionally, among the 20 participants from hospital admissions data with ICD-10 consistent with a diagnosis of APS-1 (ICD code E31.0), 18 did not have rare *AIRE* variants, and 2 had recessively inherited causative *AIRE* variants. This likely reflects the fact that ICD-10 code E31.0 captures autoimmune polyglandular failure more broadly, including Schmidt syndrome, and therefore is not specific to genetically confirmed *AIRE*-related APS-1 [24]. Individual-level details for the 2 participants with recessively inherited pathogenic *AIRE* variants cannot be reported in accordance with UK Biobank small-number reporting guidance [25].

### Domain-specific aggregated variant analyses did not identify increased disease risk

**Figure 3:**
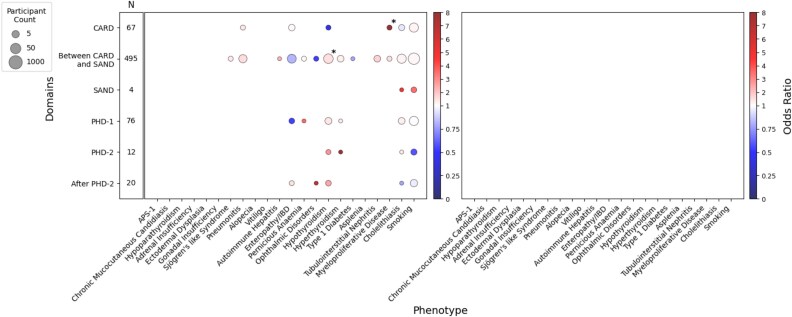
Domain-based aggregation of *AIRE* variant associations with autoimmune and related phenotypes. Left panel: Bubble plot showing the uncorrected association (OR) between aggregated *AIRE* variants grouped by their affected functional domains (rows) and phenotypes (columns). Domains include CARD, SAND, PHD1 and PHD2. Bubble size reflects the number of individuals carrying variants in each category, while colour indicates the strength and direction of the association (red = OR > 1; blue = OR < 1). Asterisks (^*^) denote nominal statistical significance (*P* < 0.05). Variants in the PHD1 domain and those spanning multiple domains are most prevalent, allowing for greater statistical power. Right panel: FDR-adjusted results for the same variant-phenotype associations. No associations remained statistically significant after multiple testing correction. Phenotypes include key autoimmune and immune-related conditions and select control traits (e.g. smoking) displayed along the x-axis. Nominal associations should be interpreted cautiously, given the absence of significance after multiple testing correction.

We next performed domain-specific analysis (Fig. 3) by grouping rare missense variants that have supportive in-silico evidence of deleteriousness, based on AlphaMissense [26], according to the functional region of the AIRE protein in which they reside (CARD, SAND, PHD1, PHD2). Despite PHD1 being reported as a hotspot for missense mutations, with 76 variant carriers of 35 variants, no statistically significant associations were found between any domain-level variant grouping and autoimmune phenotypes (Fig. 3). Two nominal associations were observed prior to correction: variants in the CARD domain were associated with an increased odds of myeloproliferative disease (OR = 11.49, 95% CI [2.81–46.98], *P* = 1.41 × 10^−3^), and variants in the region between the CARD and SAND domains were associated with an increased odds of hypothyroidism (OR = 2.73, 95% CI [1.43–5.21], *P* = 4.76 × 10^−3^). However, neither association survived FDR correction (q ≥ 0.99). These results therefore do not provide statistically significant evidence that domain-specific variant groupings increase autoimmune risk in the general UK Biobank population. These nominal signals should be interpreted with caution given the small carrier counts, broad confidence intervals, and lack of significance after multiple-testing correction.

## Discussion

After correction for multiple testing, we found no statistically significant associations between literature-reported heterozygous *AIRE* variants and autoimmune phenotypes in an unselected population, although several nominal associations were observed. Among 449 075 UKB participants, carriers of 13 of 25 literature-reported heterozygous *AIRE* variants were identified (Fig. 1), yet no associations with classical or extended autoimmune phenotypes survived multiple-testing correction (Fig. 2).

Several nominal associations were observed, including for hypoparathyroidism and hypothyroidism, but these should be interpreted cautiously given the small carrier counts, broad confidence intervals, and lack of significance after multiple-testing correction. Notably, the carrier burden was dominated by the partially inhibitory frameshift p.L323SfsTer51 (MAF = 1.39 × 10^−3^), a frequency in an unselected cohort that is incompatible with a highly penetrant pathogenic effect. The absence of association despite reported *in vitro* disruption of AIRE activity (Supplementary Table 1) is consistent with limited penetrance of heterozygous variants outside clinically ascertained cohorts and raises the possibility that some previously reported variants have little or no clinically meaningful biological effect *in vivo*, consistent with prior *in vivo* data for p.Val301Met [16].

A small subset of heterozygous *AIRE* variants with reported DN effects can cause non-classical APS-1 in families, but our population-scale analyses indicate that most heterozygous *AIRE* variants do not appear to increase autoimmune risk in unselected individuals. The lack of statistically significant associations, despite *in vitro* disruption, supports limited, context-dependent penetrance outside clinically ascertained settings and a predominantly recessive model for *AIRE*-associated autoimmunity. Clinically, incidental heterozygous *AIRE* findings should be interpreted conservatively, as domain location or in-silico evidence alone is insufficient. Population-level genomic data remain a key tool for separating *in vitro* functional disruption from clinically meaningful disease risk and should be integrated with family studies and standardised functional assays to guide variant interpretation.

We did not detect any carriers of the reported DN *AIRE* variants: p.Cys311Tyr, p.Gly228Trp and p.Cys446Gly. p.Cys311Tyr and p.Gly228Trp have prior *in vitro*, *in vivo* or familial evidence supporting DN effects and have been shown to segregate with autoimmune disease in affected families in an autosomal-dominant manner [11, 12, 14, 15]. p.Cys446Gly also has supporting *in vitro* and *in vivo* evidence, although the reported phenotype is milder and more variably penetrant [12, 16]. The absence of these variants in the UKB likely reflects the fact that these are genuinely rare, highly penetrant, monogenic causes of *AIRE*-related autoimmunity that are likely not captured in a relatively healthy volunteer cohort [27, 28].

Domain-based aggregation analyses were likewise null (Fig. 3). The PHD1 domain, which is commonly where several literature-reported heterozygous *AIRE* variants reside (e.g. p.Arg303Gln, p.Val301Met and p.Pro326Leu) has been implicated in impaired histone binding and defective multimerisation [12, 13]. However, when rare missense variants with supportive computational evidence in the PHD1 region were aggregated and analysed at the population level, no significant associations with autoimmune phenotypes were observed. We found no evidence that domain localisation alone is sufficient to infer pathogenicity for heterozygous *AIRE* variants when haploinsufficiency is not the operative mechanism.

Our findings highlight the value of large-scale studies and population-based evidence when interpreting rare variants, although they likely reflect a minimal penetrance estimate due to well-known biases toward healthy individuals [27, 28]. Guidance on penetrance estimation in population cohorts also emphasises this issue [29]. Family-based and functional studies [11–13] have established causality for many genes and variants, but such cohorts are enriched for disease and can overestimate penetrance [18, 29]; moreover, even sophisticated *in vitro* assays are imperfect proxies for complex *in vivo* biology [16, 17]. By contrast, population-scale analyses show that carriers of the same or similar variants do not consistently manifest autoimmune disease, supporting limited penetrance outside clinically ascertained families. Robust interpretation of rare *AIRE* variants therefore requires integrating functional data with familial observations and population-level effect estimates.

Despite analysing ~ 450 000 individuals, the small number of carriers for many individual variants limited statistical power to detect associations or perform adjusted analyses. This challenge is inherent to studying rare variants, even in large cohorts [30]. Additionally, several literature-reported variants were not observed in the UKB (Fig. 1), including the three *AIRE* variants with in vivo or familial co-segregation: p.Gly228Trp, p.Cys311Tyr and p.Cys446Gly. The absence of some of these variants from a large population cohort may support their pathogenicity under variant-interpretation frameworks [31, 32]. Together with the lack of association for the literature-reported variants that were observed, this suggests that UK Biobank does not harbour heterozygous *AIRE* variants with clear evidence for a strong population-level autoimmune effect. The finding that only 2 of 20 participants coded with E31.0 carried recessively inherited pathogenic *AIRE* variants also highlights a limitation of phenotyping in UK Biobank. ICD-10 code E31.0 captures autoimmune polyglandular failure more broadly, including Schmidt syndrome, and is therefore not specific to genetically confirmed *AIRE*-related APS-1. Individual-level data for the 2 participants with biallelic pathogenic *AIRE* variants cannot be disclosed because UK Biobank small-number reporting guidance restricts disclosure where counts are fewer than five [25].

Finally, we relied on electronic health record data—specifically ICD-10 codes, OPCS-4 operation/intervention codes and self-reported diagnoses, which may miss subclinical, atypical, or undiagnosed autoimmune manifestations, and can be inaccurate [33, 34]. Accordingly, our findings should be interpreted as evidence against strong population-level effects in UKB, rather than as excluding more subtle or incompletely captured phenotypes.

In conclusion, we found that rare heterozygous *AIRE* variants previously reported in association with autoimmunity and observed in UK Biobank did not show statistically significant associations with APS-1-related or other autoimmune phenotypes captured in this population after correction for multiple testing, although nominal associations were observed for some phenotypes. The identification of rare heterozygous *AIRE* variants should be interpreted with caution. Our results do not support population screening for heterozygous rare *AIRE* variants, outside the small number of variants with *in vivo* or familial support, in asymptomatic cohorts such as genomic screening programmes.

## Materials and methods

### Literature review of reported heterozygous *AIRE* variants

We conducted a systematic literature review on 10^th^ October 2025 to identify rare heterozygous *AIRE* variants reported as having dominant-negative or partially inhibitory effects and associated with autoimmune phenotypes. The PubMed database was queried using the search terms:

(‘*AIRE*’[All Fields] OR ‘autoimmune regulator’[All Fields]) AND (mutation OR variant OR missense OR genotype OR allele) AND (dominant OR heterozygous OR monoallelic OR ‘dominant-negative’) AND (autoimmune OR APECED OR ‘APS-1’ OR polyendocrinopathy)

This search yielded 116 publications. Abstracts were screened for relevance to human genetic studies reporting *AIRE* variants, with mention of heterozygous or dominant inheritance. In the full-text review, studies were included if at least one patient was reported to carry a heterozygous *AIRE* variant without other putative pathogenic variants. Studies describing compound heterozygotes, homozygotes, or cases with additional rare variants were excluded.

Papers where zygosity status or variant segregation was unclear (e.g. cases that grouped homozygous and heterozygous carriers without differentiation, or cases reported as DN but homozygous for a nonsense variant) were flagged and further reviewed.

The final set of literature-reported heterozygous *AIRE* variants identified through this process is summarised in Fig. 1 (additional information in Supplementary Table 2). Data extraction focused on variant identification, zygosity, reported phenotypic features and available functional validation (additional information in Supplementary Table 1).

### Cohort and study design

We conducted a retrospective cohort study using data from 449 075 participants in the UKB, a large-scale population resource with linked genotype and longitudinal health records [20]. All individuals included in the analysis had available whole-genome sequencing data and corresponding phenotype information derived from linked Hospital Episode Statistics (HES), baseline self-reported data and the Interventions and Procedures (OPCS-4) classification.

### Variant identification and classification

From the 17 relevant articles identified in our literature search, we identified 25 heterozygous rare *AIRE* variants previously reported as having dominant-negative or partially inhibitory effects [11, 13, 14]. We did not treat this prior description as proof of pathogenicity, but rather used these variants to define a literature-reported variant set for analysis. Of these, three variants were reported as dominant-negative, have *in vitro* and *in vivo* functional validation or familial co-segregation: NM_000383.4:c.682G > T (p.Gly228Trp) and NM_000383.4:c.932G > A (p.Cys311Tyr). In addition, NM_000383.4:c.1336 T > G (p.Cys446Gly) has supporting *in vitro* and *in vivo* evidence, although the reported phenotype is milder and has variable penetrance.

The remaining 22 variants were also considered rare heterozygous *AIRE* variants previously reported in association with autoimmunity, having been described in the literature as DN-like or as having partially inhibitory effects in functional assays but with variable penetrance and expressivity in reported carriers. Within this group, three variants showed only partial inhibition of wild-type AIRE activity: NM_000383.4:c.967_979del13bp (p.L323SfsTer51), NM_000383.4:c.946C > T (p.Arg316Trp) and NM_000383.4:c.983G > A (p.Arg328Gln). Whilst we note that NM_000383.4:c.901G > A (p.Val301Met) did not affect mTEC expression in a mouse knock-in model [16], it has continued to appear in more recent reports of autoimmune phenotypes in heterozygous carriers [13]. These literature-derived definitions were used for downstream analyses (see Supplementary Table 2).

Variant data were obtained from UKB whole-genome sequencing (PLINK format) and annotated using the Ensembl Variant Effect Predictor (VEP) [35]. In addition to specifically identifying literature-reported heterozygous *AIRE* variants, we filtered and aggregated coding variants by rarity (Minor Allele Frequency [MAF] < 0.001) and consequence, to identify potential additional variants of interest. Coding masks included frameshift/protein-truncating variants (PTVs) [excluding those in terminal exon or last 50 bp of penultimate exon, and therefore expected to result in nonsense-mediated decay and loss of the allele], missense, in-frame indels and splice-site variants (canonical and non-canonical), with the remaining cohort used as a reference group. When prioritising missense variants, we required supportive in-silico evidence based on AlphaMissense, using a threshold consistent with ACMG/AMP PP3-supporting computational evidence [26]. We filtered individual-level genotypes based on a minimum sequencing depth of 15 reads, genotype quality ≥30, and allelic balance ≥30% (Fig. 1). We also performed manual IGV checks on all frameshift/PTVs and in-frame indels to ensure they were called correctly [36]. Variants with MAC < 10 were collapsed into an ultra-rare group, exceeding UKB small-number disclosure thresholds (presence in < 5 participants) [25].

Based on prior evidence of domain clustering among literature-reported heterozygous *AIRE* variants [13], we sought to explore these effects by grouping variants by protein domain localisation—specifically the CARD, PHD1, PHD2 and SAND domains (see Supplementary Table 2). For external comparison and validation, aggregated gene-level findings were cross-referenced with publicly available phenome-wide association resources (AZPheWAS [21] and GeneBass [22]).

### Phenotype definitions

Phenotypes were selected based on clinical features associated with APS-1, ensuring comprehensive coverage of classical, non-classical, and broader autoimmune manifestations previously reported in APS-1 literature. Phenotypes included major APS-1 components (e.g. Addison’s disease, hypoparathyroidism, chronic mucocutaneous candidiasis) [3, 4, 37, 38], features commonly associated with APS-1 (e.g. gonadal insufficiency, vitiligo, autoimmune hepatitis) [5, 10] and additional traits described in APS-1 clinical case reports, including pneumonitis, tubulointerstitial nephritis, and Sjögren’s-like syndrome [7, 38]. The complete list of phenotypes and corresponding codes is provided in Supplementary Table 3.

For each phenotype, we excluded drug-induced, post-procedure, or otherwise non-autoimmune analogues to maximise specificity (e.g. steroid-induced adrenal insufficiency; post-thyroidectomy hypoparathyroidism; non-APS-1 candidiasis related to acute infection). Exclusions were implemented using explicit ICD-10 exclusion codes and, where relevant, OPCS-4 procedure codes (e.g. adrenalectomy, thyroidectomy; full code lists in Supplementary Table 2).

For type 1 diabetes, which is difficult to accurately define in population databases due to miscoding with much more common type 2 diabetes, we applied established genetic and clinical criteria [39]. Smoking status, used as a control for phenotype specificity, was defined as ‘ever smoker’ (comprising current and former smokers) versus ‘never smoker’ according to responses at the initial assessment visit (Field 20 116) [40, 41].

Phenotype data were derived from ICD-10 diagnostic codes, OPCS-4 operation/procedure codes and self-reported questionnaire data collected at UKB baseline recruitment. All phenotype information was sourced from the UKB-linked HES and baseline self-report fields.

### Statistical testing

After identifying variant carriers and categorising them by variant effect, protein domain localisation, and obtaining associated phenotype counts, we conducted a series of Fisher’s exact tests to determine whether the prevalence of specific phenotypes differed significantly between each variant group (by consequence or domain) and the remainder of the cohort. For each comparison, we calculated odds ratios to estimate the magnitude and direction (risk or protection) of any observed associations. Multiple testing correction was performed using a 5% Benjamini–Hochberg False Discovery Rate (FDR) threshold to account for the large number of comparisons and reduce the likelihood of false-positive findings. Associations were considered significant only if they passed this FDR threshold.

## Supplementary Material

Supplementary_figures_ddag047

Supplementary_materials_ddag047

Supplementary_Table_2_ddag047

## Data Availability

This research has been conducted using the UK Biobank Resource under Application Number 103356. UKB does not allow patient-level data to be dispensed. Additional summary statistics are available from the authors upon reasonable request.

## Code Availability

Code for variant annotation, filtering, and analysis is available at https://github.com/Surajram112/AIRE.
