# Supplementary material for: Limited penetrance of dominantly inherited AIRE variants in a population-based cohort
Source: Hum Mol Genet. 2026 Jun 9;35(11):ddag047. doi: 10.1093/hmg/ddag047 (PMC13248555; doi:10.1093/hmg/ddag047)
Supplement: Supplementary_figures_ddag047 [file supplementary_figures_ddag047.docx]

**Supplementary Figures**

UKB Cohort with

HES Records

**N=449075**

Ultra Rare

N=12

V=4

Non-Carriers:

N = 444450

Single Variants

N=2111

V=9

Literature-reported

N=2123

V=13

Rare Het *AIRE* Variants:
N=4057

V= 163

Other

N=1934

V=153

Quality Control:

Depth >= 15x

Genotype Quality >= 30

Min Allelic Balance>= 30%

**Supplementary Figure 1:** Variant Stratification and Cohort Breakdown of *AIRE* Variant Carriers in the UK Biobank


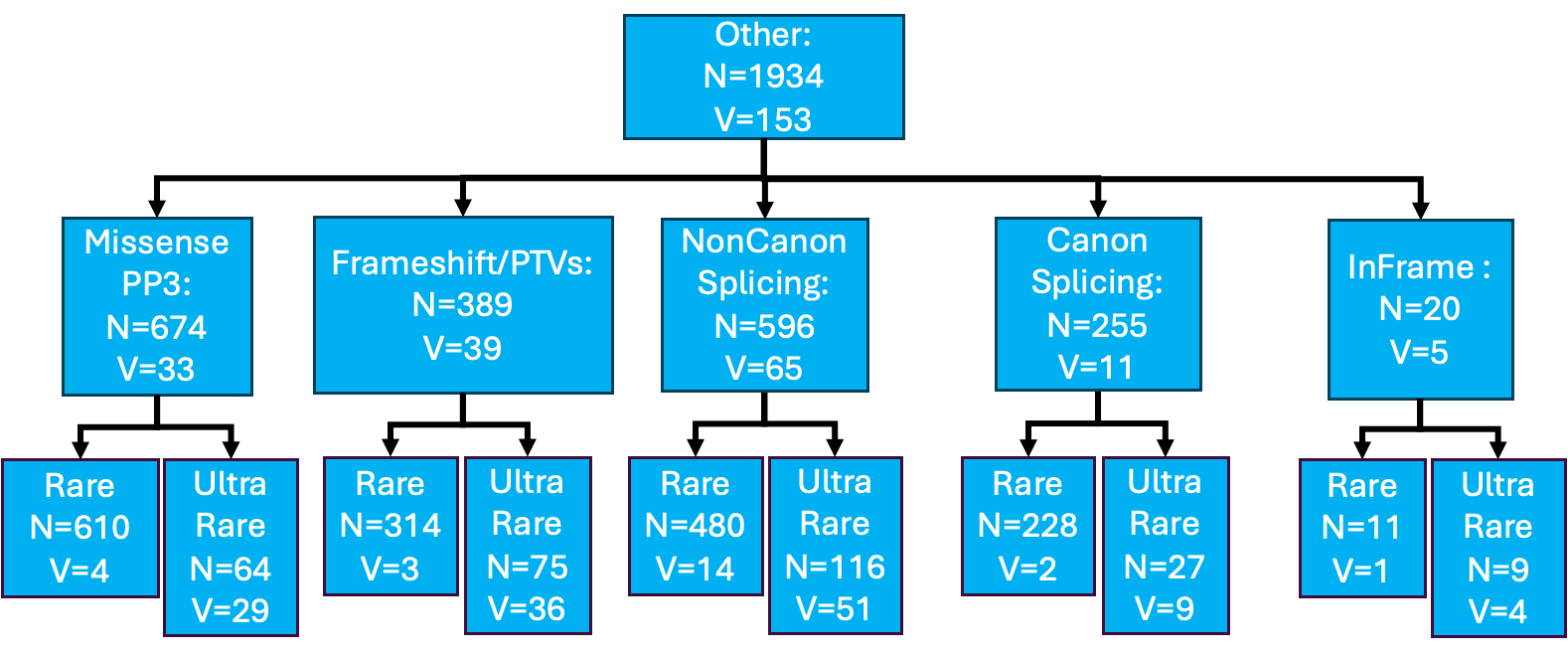
Flowchart illustrating the classification of participants from the UK Biobank cohort with linked Hospital Episode Statistics (HES) data, stratified by carrier status for rare heterozygous *AIRE* variants analysed in this study. Initially, quality control, including sequencing depth ≥15, genotype quality ≥30, and minimum allelic balance ≥30%, were applied across all the extracted variants. Following QC, individuals were grouped into non-carriers and carriers of variants predicted to be pathogenic. Among carriers, variants were further categorised into previously reported dominant-negative mutations and other potentially pathogenic variants. Dominant-negative variants were subdivided by occurrence pattern (e.g., single variants, ultra rare), and all variants were classified by rarity (ultra rare vs. rare) for downstream analyses. The number of individuals (N) and unique variants (V) are indicated at each stage.

**Supplementary Figure 2:** Breakdown of other rare heterozygous *AIRE* Variants in UK Biobank

Flowchart depicting the categorisation of *AIRE* variants classified as other rare coding *AIRE* variants and broken down into functional consequence: missense, frameshift/protein truncating variants (PTVs), non-canonical splicing, and in-frame variants, with subdivisions to further stratified by rarity (ultra rare vs. rare). The number of unique variants (V) and the number of individuals carrying them (N) are indicated.
