# Supplementary material for: Limited penetrance of dominantly inherited AIRE variants in a population-based cohort
Source: Hum Mol Genet. 2026 Jun 9;35(11):ddag047. doi: 10.1093/hmg/ddag047 (PMC13248555; doi:10.1093/hmg/ddag047)
Supplement: Supplementary_materials_ddag047 [file supplementary_materials_ddag047.zip › Nomenclature Statement.docx]

**Nomenclature Statement**

All gene and variant descriptions in this manuscript adhere to the HUGO Gene Nomenclature Committee (HGNC) and the **Human Genome Variation Society (HGVS)** nomenclature guidelines. The transcript used is the *AIRE* **RefSeq canonical transcript (NM_000383.4)**.

The following illustrates the variants studied and their HGVS and Hg38 nomenclature:

| **Coding DNA (HGVSc)** | **Protein sequence (HGVSp)** | **Genomic (HGVSG; Hg38)** |
| --- | --- | --- |
| **NM_000383.4** | **NP_000374.1** | **NC_000021.9** |
| c.967–979del13bp | p.(Leu323Serfs*51)  p.(L323SfsTer51) | g.44291182_44291194del |
| c.901G>A | p.(Val301Met)  p.(V301M) | g.44291116G>A |
| c.982C>T | p.(Arg328Trp)  p.(R328W) | g.44291197C>T |
| c.908G>A | p.(Arg303Gln)  p.(R303Q) | g.44291123G>A |
| c.1399G>C | p.(Gly467Arg)  p.(G467R) | g.44293909G>C |
| c.977C>T | p.(Pro326Leu)  p.(P326L) | g.44291192C>T |
| c.946C>T | p.(Arg316Trp)  p.(R316W) | g.44291161C>T |
| c.739C>T | p.(Arg247Cys)  p.(R247C) | g.44289743C>T |
| c.1102C>G | p.(Pro368Ala)  p.(P368A) | g.44292999C>G |
| c.983G>A | p.(Arg328Gln)  p.(R328Q) | g.44291198G>A |
| c.892G>A | p.(Glu298Lys)  p.(E298K) | g.44291107G>A |
| c.913G>A | p.(Gly305Ser)  p.(G305S) | g.44291128G>A |
| c.607C>T | p.(Arg203Ter)  p.(R203X) | g.44288413C>T |
| c.622G>T | p.(Gly208Trp)  p.(G208W) | g.44288428G>T |
| c.682G>T | p.(Gly228Trp)  p.(G228W) | g.44289686G>T |
| c.890A>C | p.(Asp297Ala)  p.(D297A) | g.44291105A>C |
| c.905G>A | p.(Cys302Tyr)  p.(C302Y) | g.44291120G>A |
| c.908G>C | p.(Arg303Pro)  p.(R303P) | g.44291123G>C |
| c.916G>A | p.(Gly306Arg)  p.(G306R) | g.44291131G>A |
| c.926T>C | p.(Ile309Thr)  p.(I309T) | g.44291141T>C |
| c.932G>A | p.(Cys311Tyr)  p.(C311Y) | g.44291147G>A |
| c.934G>A | p.(Asp312Asn)  p.(D312N) | g.44291149G>A |
| c.1010G>T | p.(Cys337Phe)  p.(C337F) | g.44292316G>T |
| c.1024C>T | p.(Gln342Ter)  p.(Q342X) | g.44292330C>T |
| c.1336T>G | p.(Cys446Gly)  p.(C446G) | g.44293846T>G |
